# Next generation sequencing unravels the biosynthetic ability of Spearmint (*Mentha spicata*) peltate glandular trichomes through comparative transcriptomics

**DOI:** 10.1186/s12870-014-0292-5

**Published:** 2014-11-01

**Authors:** Jingjing Jin, Deepa Panicker, Qian Wang, Mi Jung Kim, Jun Liu, Jun-Lin Yin, Limsoon Wong, In-Cheol Jang, Nam-Hai Chua, Rajani Sarojam

**Affiliations:** Temasek Life Sciences Laboratory, 1 Research Link, National University of Singapore, Singapore, 117604 Singapore; School of Computing, National University of Singapore, Singapore, 117417 Singapore; Laboratory of Plant Molecular Biology, The Rockefeller University, 1230 York Avenue, New York, NY 10065 USA; Department of Biological Sciences, National University of Singapore, Singapore, 117543 Singapore

**Keywords:** Spearmint, Next generation sequencing, Transcriptome, Glandular trichomes, Terpenes, Carvone, Terpene synthases

## Abstract

**Background:**

Plant glandular trichomes are chemical factories with specialized metabolic capabilities to produce diverse compounds. Aromatic mint plants produce valuable essential oil in specialised glandular trichomes known as peltate glandular trichomes (PGT). Here, we performed next generation transcriptome sequencing of different tissues of *Mentha spicata* (spearmint) to identify differentially expressed transcripts specific to PGT. Our results provide a comprehensive overview of PGT’s dynamic metabolic activities which will help towards pathway engineering.

**Results:**

Spearmint RNAs from 3 different tissues: PGT, leaf and leaf stripped of PGTs (leaf-PGT) were sequenced by Illumina paired end sequencing. The sequences were assembled *de novo* into 40,587 non-redundant unigenes; spanning a total of 101 Mb. Functions could be assigned to 27,025 (67%) unigenes and among these 3,919 unigenes were differentially expressed in PGT relative to leaf - PGT. Lack of photosynthetic transcripts in PGT transcriptome indicated the high levels of purity of isolated PGT, as mint PGT are non-photosynthetic. A significant number of these unigenes remained unannotated or encoded hypothetical proteins. We found 16 terpene synthases (TPS), 18 cytochrome P450s, 5 lipid transfer proteins and several transcription factors that were preferentially expressed in PGT. Among the 16 TPSs, two were characterized biochemically and found to be sesquiterpene synthases.

**Conclusions:**

The extensive transcriptome data set renders a complete description of genes differentially expressed in spearmint PGT. This will facilitate the metabolic engineering of mint terpene pathway to increase yield and also enable the development of strategies for sustainable production of novel or altered valuable compounds in mint.

**Electronic supplementary material:**

The online version of this article (doi:10.1186/s12870-014-0292-5) contains supplementary material, which is available to authorized users.

## Background

Plants produce an enormous variety of specialised metabolites among which terpenes are the largest and most structurally diverse class of natural products. They are the main components of plant essential oils. Many of these terpenes are produced and stored in specialised secretory structures called glandular trichomes [1,2]. These terpenes provide protection for plants against a variety of herbivores and pathogens [3] and are also commercially quite valuable. Therefore, the processes by which they are synthesised and stored in plants are main target for genetic manipulation for increased yield. But our knowledge about the development of secretory glandular trichomes and terpene production and its regulation is very limited making it difficult to engineer these metabolic pathways [4,5].

Aromatic essential oil produced by *Mentha* species is the source of the best known monoterpenes, menthol and carvone, which form the principal components of mint oil. They are extensively used in flavour and fragrance industries, pharmaceuticals and cosmetic products [6]. Peppermint variety mostly produces menthol whereas in spearmint variety carvone dominates [7,8]. From the PGT of peppermint variety ( *Mentha X piperita*), 1,316 randomly selected cDNA clones, or expressed sequence tags (ESTs) were produced, which led to the identification of many genes, enzymes and substrates involved in the main menthol essential oil biosynthetic pathway [9,10]. Given the technical limitations at their time of study, an EST approach would possibly identify only cDNAs which are abundant in PGT. A recent proteomic analysis of spearmint PGT identified 1,666 proteins of which 57 were predicted to be involved in secondary metabolism [11]. But generation of sufficient genomic information with deep coverage is required to gain insights into the regulatory mechanism of terpene metabolism and glandular trichome development. This will promote successful engineering for improved yields or to develop mint as a platform for production of novel/altered terpenes. Mint is a well-suited plant for this as it is able to produce and store large amount of oils within PGT instead of exuding it on to the leaf surface. Storage within the PGTs also reduces the loss of volatile oils by emission into the atmosphere.

High-throughput RNA sequencing (RNA-Seq) has increasingly become the technology of choice to generate a comprehensive and quantitative profile of the gene transcription pattern of a tissue. Here, we report comparative analysis of RNA-seq transcriptome of different tissues of spearmint-namely PGT, leaf minus PGT (leaf-PGT) and leaf. The transcriptome data provided a genome-wide insight into the metabolic ability of PGT. Comparison of PGT and leaf-PGT showed that 3,919 unigenes were differentially expressed in PGT (minimum 4 times more in PGT when compared to leaf -PGT). Many of these were related to terpene production and other secondary metabolite pathways. From the various terpene synthases (TPS) transcripts identified, we functionally characterized 2 of these previously uncharacterized TPSs from mint and found them to be sesquiterpene synthases. Key pathway unigene transcripts were verified by qRT-PCR. Our results show the molecular specialisation of PGT for the production of different classes of metabolites.

## Results and discussion

### Spearmint PGT and their development

**Figure 1:**
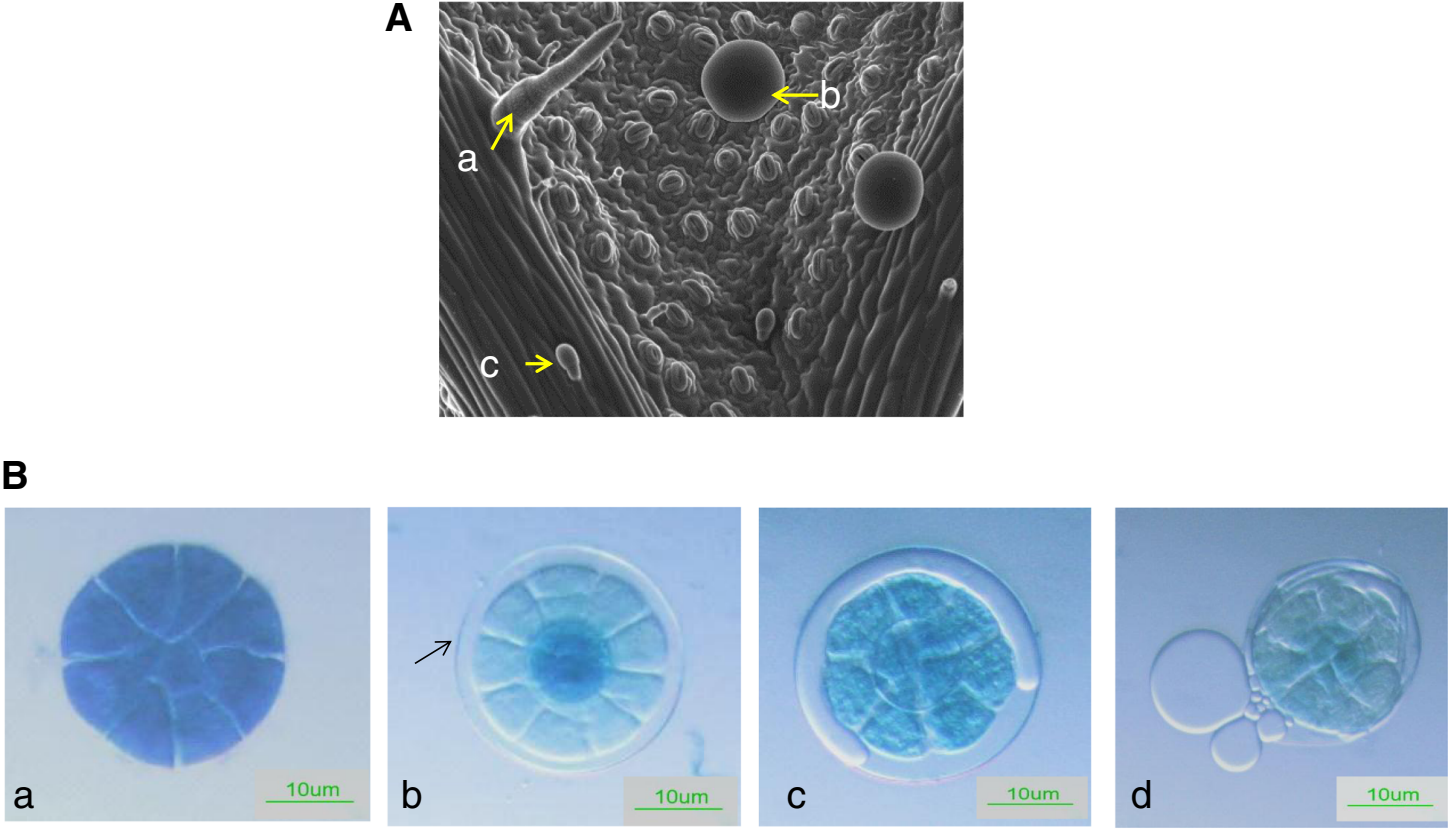
**Trichomes on spearmint leaf. (A)** Scanning electron microscope image of spearmint leaf showing three types of trichomes, **a**, Non glandular hairy trichome; **b**, Peltate glandular trichome (PGT); **c**, Capitate glandular trichome. **(B)** Process of secretion by PGT. **a**, presecretory stage; **b**, formation of storage cavity; **c**, secretion into the storage cavity; **d**, release of oil upon injury. The PGTs were stained with toulidine blue.

**Figure 2:**
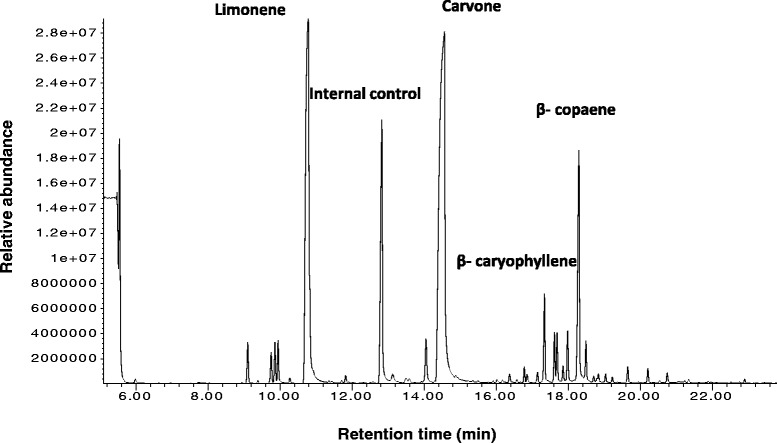
**GC-MS of spearmint leaf showing the presence of monoterpenes and sesquiterpenes.**

Spearmint leaves produce three different types of trichomes on their surfaces: non-glandular multicellular hair like, capitate glandular trichomes with a single secretory head cell and PGTs whose secretory head is composed of eight-cells with a single stalk and basal cell (Figure 1A). These PGT glands possess a large subcuticular storage space that is formed by the separation of the cuticle from the apical cells and the essential oil is secreted into this cavity [12] (Figure 1B). It is known that new glands keep initiating on the leaf till expansion ceases and the monoterpene content and compositions change with the age of the leaf [13-16]. Different studies have indicated that monoterpene biosynthesis is most active in young 12–20 day old leaves of peppermint after which the rate of synthesis slowly declines [17-19]. We performed gas chromatography–mass spectrometry (GC-MS) analysis on young spearmint leaves (about 1–2 cm in length) and found abundance of both limonene and carvone monoterpenes (Figure 2). Limonene is the first committed step towards carvone pathway. In addition to these monoterpenes, the presence of sesquiterpenes was also observed. This indicated the dynamic terpene biosynthetic activity of leaves at this stage of development. PGT were purified from leaves of this stage and RNA isolated. The leaves of the same stage were brushed to remove all trichomes and RNA extracted from them as controls (Additional file 1).

### Sequencing, *de novo* assembly and annotation of transcriptome

Three RNA libraries were prepared and sequenced by Illumina technology. More than 100 million high quality reads of 101 base pairs (bp) were generated from PGT, leaf-PGT and leaf (Additional file 2). Using the Trinity method [20] the sequence reads were finally assembled into 40,587 non-redundant unigenes, spanning a total of 101 Mb of sequence with a GC content of 43.14%. All unigenes were longer than 200 bp. The N50 of the final assembled transcripts was 1,774 bp. The unigenes were annotated by performing BLASTX search against various protein databases. Among the 40,587 non-redundant unigenes, 27,025 (67%) had at least one hit in BLASTX search with E-value < = 1e-3. Functional classifications of Gene Ontology (GO) term of all unigenes were performed using Trinotate [20]. In order to calculate the expression level for assembled transcripts, we first mapped reads onto them using bowtie [21]. RSEM (RNA-seq by Expectation-Maximization) was used to estimate the abundance of assembled transcripts and to measure the expression level [22].

### Overview of expression profile of spearmint PGT

**Figure 3:**
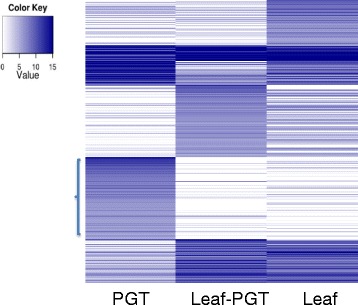
**Heat map of transcript expression in PGT, leaf-PGT and leaf.**

From the RNA seq data about 25,000 unigenes were observed to be expressed in spearmint PGT. The heat map in Figure 3 exhibits some specific expression patterns to PGT. Among this specific pattern for PGT we found transcripts for terpene biosynthesis, lipid transfer proteins and interesting transcription factors like MYBs and WRKYs. Comparison of PGT and leaf-PGT showed that 3,919 unigenes were differentially expressed in PGT (Additional file 3). These unigenes showed a minimum of 4 times increase in expression level in PGT as compared to leaf-PGT. About 30% of these unigenes encoded either hypothetical proteins or remained unannotated. Many of these unannotated unigenes showed none or minimal expression in leaf-PGT. They might represent novel genes that are unique to PGTs development and divergent from other plants, whose genomes have been sequenced. Data from proteomic analysis of spearmint PGT also showed that the largest functional category of the identified proteins was “unclear classification” and included proteins with unknown functions [11]. The absence or low levels of some PGT-specific transcripts from leaf RNA seq data indicated the dilution of PGT-specific RNAs among the total leaf RNAs and reaffirms the importance of isolating these organs for analysis.

Among the top 1000 differentially expressed unigenes, we identified 16 TPSs, 18 cytochrome P450s, 5 lipid transfer proteins (LTPs), 20 transcription factors, 2 ATP-binding cassette (ABC) transporters and several transcripts associated with cell wall. Cytochrome P450s are involved in the hydroxylation of terpenes [23] and LTPs have been suggested to be involved in intracellular transport and secretion of lipids and terpenes [9,24]. LTPs were among the most abundant unigenes in PGT and were confirmed by qRT-PCR (Additional file 4). The abundance of these LTPs suggests their importance in PGTs metabolic function and development. ABC transporters are also proposed to be involved in the active transport of secondary metabolites [11,25]. The spearmint PGT is presumed to undergo cell wall modification to form subcuticular storage space [12]. Among the differentially expressed unigenes there were few that were related to cell wall synthesis or modifications and a subset of these were confirmed by qRT-PCR (Additional files 4 and 5). Whether they play a role in modification of cell wall layers to form the storage space remains to be investigated.

**Figure 4:**
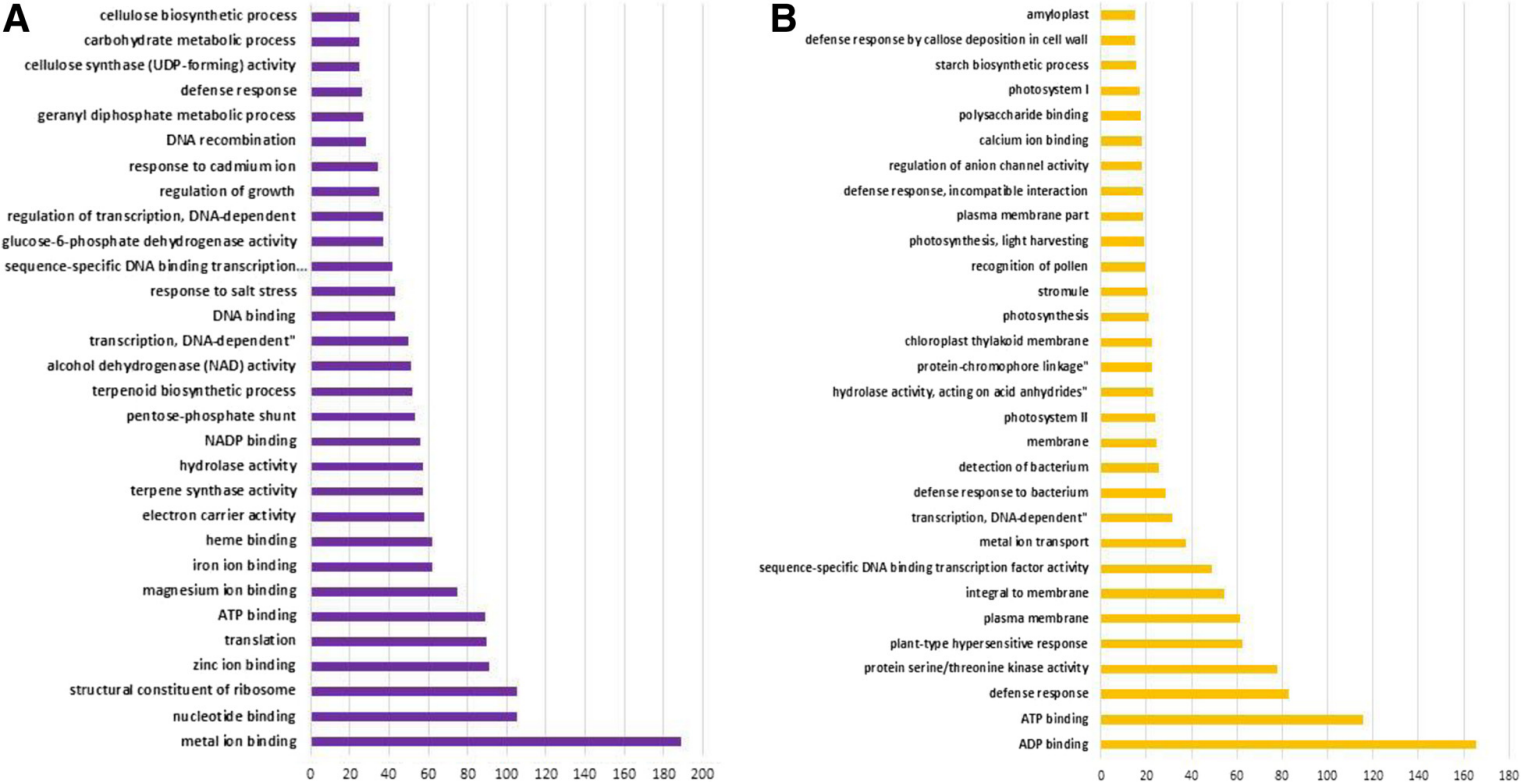
**Top 30 GO annotation of more expressed unigenes (A) and less expressed unigenes (B).** X-axis: log(1/P-value), P-value is the hypergeometic test result for each GO terms.

To characterize biological processes specific for PGT, GO term was determined for all differentially expressed unigenes. Additionally, we identified unigenes whose expression was reduced in PGT by comparing leaf-PGT and PGT. These unigenes showed a minimum of 4 times reduction in expression level in PGT when compared to leaf-PGT. GO term was determined for them as well. Figure 4 shows the top 30 GO terms for the more abundant and less abundant unigenes. GO terms associated with ribosome biogenesis, ribosome structural genes and translation are highly represented in PGT, which could reflect the high protein biosynthetic activity of PGTs. Other terms included terpene metabolism and most of the primary energy producing terms like glycolysis and tricarboxylic acid cycle. Furthermore, pentose phosphate related term (oxidative) was also enriched in PGT. This term provides NADPH for biosynthetic processes such as fatty acid synthesis, cytochrome P450 mediated hydroxylation and the assimilation of inorganic nitrogen [26]. These results indicate that the GO functions that provide energy equivalents and redox cofactors are very active in PGT. Secretory trichomes are biosynthetically very active, and hence, there is a high energy requirement in these cells. Unigenes from the GO terms of photosynthesis, chlorophyll biosynthesis and starch biosynthesis were among the less abundant ones. This shows that our PGT sample preparation was pure and not contaminated with leaf tissues as mint PGTs are non-photosynthetic.

Mint PGTs being non- photosynthetic and metabolically very active would presumably rely on exogenous supply of sucrose from underlying leaf tissues to use as carbon source for energy production. We found several transcripts encoding enzymes for sucrose catabolism expressed more in PGT like sucrose synthase and neutral/alkaline invertases that are important for channelling carbon from sucrose in non-photosynthetic tissues [27]. These enzymes convert sucrose to hexose phosphates. Plastids in the PGTs are the main sites of secondary metabolism. In contrast to chloroplasts, plastids of heterotrophic tissues have to rely on the import of ATP and carbon to drive their metabolic processes. We checked our set of differentially expressed unigenes to see if any known transporters are present. In most plants glucose 6-phosphate seems to be the preferred hexose phosphate taken up by nongreen plastids. The transporter proteins responsible for this import of carbon into plastids are known as Glc6P–phosphate translocator (GPT) and transcript similar to GPT was seen enriched in PGT (about 30 times more in PGT). This carbon can be used for starch biosynthesis or for the oxidative pentose phosphate pathway in plastids [28]. GO terms for oxidative pentose phosphate pathways were seen enriched in PGT. Additionally transcript similar to plastidic Phosphoenolpyruvate/phosphate translocator was found to be expressed more in PGT. They are involved in the transport of phosphoenolpyruvate, an energy rich glycolytic intermediate from the cytoplasm into the plastids [29]. Further, ATP generated either by glycolysis or by oxidative phosphorylation in mitochondria can be imported into non- green plastids by plastidic nucleotide transporter (NTT). Transcript similar to NTT was also observed to be more abundant in PGT [29].

### Analysis of spearmint PGT transcription factors

Although the cloning and functional characterization of enzymes involved in terpene biosynthesis has been quite successful in various plants, knowledge about regulation of these secretory trichome specific pathways is very rudimentary. Studies in peppermint show a close association between enzyme activity and transcript abundance for all gene/enzyme pairs, suggesting that, essential oil biosynthesis is primarily influenced at the transcriptional level [12,17,18,30]. Hence, identification of transcription factors that globally control metabolic pathway will provide an attractive strategy for engineering terpene production. Similarly, knowledge about the development of secretory glandular trichomes, the so-called factories of important terpenoid production, is very limited. Most of the transcription factors involved with trichome development have been isolated from Arabidopsis, which lacks secretory trichomes. Studies in tobacco and tomato are beginning to show that multicellular secretory trichomes and unicellular trichomes of Arabidopsis are not homologous structures, and they likely develop under different regulatory conditions [31]. Our analysis of transcriptome data of young leaves or PGT did not uncover any transcripts that matched those of the major known trichome initiating gene transcripts from Arabidopsis, like *TRANSPARENT TESTA GLABRA1, GLABRA1, GLABRA3* [32]. Either these genes are not expressed or are expressed at a different developmental stage of leaves or PGT than the stage used in this study. Table 1 shows the top 20 transcription factors that were significantly more abundant in PGT when compared to leaf-PGT.

**Table 1:** **Top 20 enriched TFs in PGT compared to leaf-PGT**

**Name**	**Leaf**	**Leaf-PGT**	**PGT**	**FC**	**Arabidopsis ID**	**Description**
comp26629_c1	4.86	1.10	7.57	6.48	AT1G48000	myb
comp29031_c0	7.08	4.51	10.93	6.42	AT2G26580	YABBY
comp37102_c0	2.04	0.00	6.26	6.26	AT2G46150	Late embryogenesis abundant
comp31772_c0	2.48	0.10	6.03	5.93	AT5G13080	WRKY
comp31929_c0	3.97	1.52	7.27	5.75	AT2G47460	myb
comp25071_c0	3.11	1.24	6.93	5.69	AT5G60910	MADS-box
comp34871_c0	2.51	0.00	5.34	5.34	AT3G56850	Basic region/leucine zipper motif
comp34759_c0	1.69	0.00	5.25	5.25	AT1G14600	Homeodomain-like
comp43497_c1	2.42	0.53	5.36	4.84	AT1G68150	WRKY
comp32776_c1	0.70	0.00	4.61	4.61	AT2G41690	Heat shock TF
comp27387_c0	0.00	0.00	3.91	3.91	AT5G07680	NAC
comp35105_c0	2.06	0.00	3.85	3.85	AT5G65790	myb
comp25286_c0	2.13	0.00	3.80	3.80	AT5G01380	Homeodomain like TF
comp30569_c1	2.32	1.08	4.85	3.77	AT5G66350	Lateral root primordium
comp26625_c0	2.15	1.37	5.04	3.67	AT1G75390	bzip domain
comp17332_c0	0.00	0.00	3.65	3.65	AT1G73230	btf3
comp32174_c0	3.56	2.22	5.70	3.48	AT5G13080	WRKY
comp28852_c0	0.95	0.00	3.32	3.32	AT2G17770	Basic region/leucine zipper motif
comp29336_c0	3.03	1.18	4.39	3.21	AT3G24860	Homeodmain-like
comp42078_c1	3.48	0.77	3.84	3.08	AT4G32730	Homeodomain

The value is log2 RESM value for each assembled TFs. FC is the log 2 fold change in PGT when compared to leaf-PGT. Arabidopsis ID is the homolog ID in Arabidopsis protein database.

### The MEP (2-Cmethyl-D-erythritol-4-phosphate) pathway is more abundant in spearmint PGT than the MVA (mevalonate) pathway

**Figure 5:**
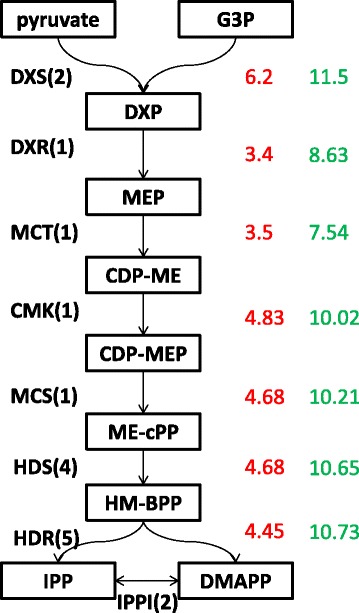
**Expression level of unigenes involved in MEP pathway.** The number in green represents the expression level of a particular unigene in PGT (log2 of estimate abundance of transcripts by RSEM value). The number in red represents the fold change in expression level when compared to leaf-PGT (log2 fold change between PGT and leaf-PGT). In cases of enzymes with more than one unigene, the unigene with the highest abundance was taken into consideration. The number in brackets represents the number of unigenes identified for each enzyme in the pathway. DXS: 1-deoxy-D-xylulose-5-phosphate (DXP) synthase; DXR: DXP reductoisomerase, MCT:MEP cytidyltransferase, CMK:4-(cytidine 5- diphospho)-2-*C*-methyl-D-erythritol kinase MCS: 2-C-methyl-D-erythritol 2,4-cyclodiphosphate (ME-2,4cPP) synthase, HDS: 1-hydroxy-2-methyl-2-butenyl 4-diphosphate (HMBPP) synthase, HDR: HMBPP reductase, IPPI : Isopentenyl diphosphate (IPP,C5) Delta-isomerase.

The building blocks for all different classes of terpenes produced by plants are C5 units of isopentenyl diphosphate (IPP) and its allylic isomer dimethylallyl diphosphate (DMAPP). They are generated either by plastidial MEP or cytoplasmic MVA pathway. The MEP pathway requires seven enzymes to synthesize IPP and DMAPP from pyruvate and glyceraldehyde 3 phosphates which feed the monoterpene pathway [33]. From peppermint EST studies it has been proposed that the active pathway for the formation of IPP/DMAPP in the PGT is the MEP pathway. This is consistent with our analysis too where MEP pathway transcripts were more abundant in PGT than MVA. High expression of MEP pathway transcripts correlates well with the production of monoterpenes in PGT. It has been reported that 1-deoxy-D-xylulose-5-phosphate synthase (DXS), the first enzyme of this pathway is important for the overall regulation of the pathway [34]. Multiple *DXS* genes have been found in plants like *Zea mays*, *Medicago truncatula, Oryza sativa, Ginkgo biloba* and *Pinus densiflora* and *Picea abies* [35-40]. In all these plants, two or three candidate *DXS* genes have been reported. From our data we were able to identify 2 different 1-deoxy-D-xylulose-5-phosphate synthase (DXS) unigenes showing different levels of abundance in PGT. The number of genes coding for each MEP pathway enzyme varies from plant to plant [33,41]. Presence of multiple genes with differential tissue-specific expression levels might contribute towards the regulation of the MEP pathway, in different organs of the plant. Figure 5 shows the number of unigenes identified for each enzyme of the MEP pathway and their RNA seq expression levels. In cases of enzymes with more than one unigene, the unigene with the highest abundance in PGT was taken into consideration. Their expression was further validated by qRT-PCR (Additional file 4). From our RNA seq data and qRT-PCR analysis, DXR and MCT transcript levels were low when compared to levels of other enzymes in MEP pathway. This might suggest that possibly these two enzymes are the rate limiting steps of this pathway. A possible option to explore in future will be to enhance the expression level of various rate limiting steps to enhance the production of terpenes.

In contrast to the MEP pathway, the transcript levels of MVA enzymes were very low. For RNA seq expression levels of unigenes involved in this pathway refer Additional file 6. Their expression was validated by qRT-PCR (Additional file 4). MVA pathway derived IPP is generally believed to be used for the production of cytosolic sesquiterpenes, triterpenes and mitochondrial terpenes. In addition to monoterpenes, mint produces a few sesquiterpenes although at much lower quantities than monoterpenes [9,42]. Lower level of MVA pathway can be one of the reasons. Interestingly, the transcripts of genes involved in the MEP pathway are also enriched in *Artemisia annua* trichomes, glandular trichomes of Hops and in Snapdragon flowers where sesquiterpene metabolism dominates [43-45] suggesting that the MEP pathway can also feed sesquiterpene production. Studies with labelled substrate predict exchange of metabolites between the MVA and MEP pathways [46,47]. How the IPP/DMAPP formed by MEP pathway is utilized to synthesize sesquiterpenes in mint remains to be investigated.

### Monoterpene production is enriched in spearmint PGT

**Figure 6:**
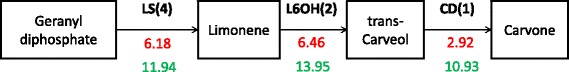
**Carvone biosynthesis pathway unigene levels.** The number in green represents the expression level of a particular unigene in PGT (log2 of estimate abundance of transcripts by RSEM value). The number in red represents the fold change in expression level when compared to leaf-PGT (log2 fold change between PGT and leaf-PGT). In cases of enzymes with more than one unigene, the unigene with the highest abundance was taken into consideration. The number in brackets represents the number of unigenes identified for each enzyme in the pathway. LS: Limonene synthase, L6OH: Limonene-6-hydroxylase, CD: Carveol dehydrogenase.

Subsequent condensation reactions between IPP and DMAPP are catalysed by GPP synthases (GPPS) that leads to the formation of geranyl diphosphate (GPP; C10) the precursor for monoterpenes. The conversion of IPP to DMAPP and its equilibrium is maintained by IPP isomerase (IPPI). In most plant species this enzyme is encoded by a single gene whereas Arabidopsis has two IPPI genes [33]. We found 2 IPPI unigenes in spearmint both enriched in PGT. Peppermint GPPS (Mp GPPS) is a two-component heteromeric enzyme consisting of a large and a small subunit, and both the subunits are catalytically inactive by themselves [48,49]. In spearmint too, we found unigenes for both the small and large subunits of GPP synthase that showed high expression in PGT. The major constituent of spearmint essential oil is (−) carvone which is synthesised from GPP in a three step reaction. Transcripts for all the three enzymes involved in above reaction, Limonene synthase (LS), Limonene-6-hydroxylase (L6OH) and carveol dehydrogenase (CD) were highly expressed in PGT and verified by q-RT-PCR (Figure 6 and Additional file 4).

Interestingly, the precursor for menthol in peppermint and (−) carvone in spearmint is the same limonene. In peppermint it is oxygenated by (2)-4S-limonene-3-hydroxylase (L3OH) to form (2)-trans-isopiperitenol and it enters the menthol pathway whereas in spearmint limonene is oxygenated by (2)-4S-limonene- 6-hydroxylase (L6OH) to form (2)-trans-carveol. Both these enzymes show a 70% identity at the amino acid level with major differences localized to the presumptive active sites [50]. The spearmint L6OH transcript is highly expressed in PGT as expected but the full set of downstream redox enzymes isopiperitenone reductase, (+)-pulegone reductase, and menthone reductase involved in menthol pathway were also found but poorly expressed in PGT. A previous study has shown that (−) carvone is not an efficient substrate for the initial double-bond reductase, therefore (−) carvone accumulates in spearmint even though the downstream redox enzymes are present [11,51]. Hence, the abundance of a single enzyme L6OH instead of L3OH changes the final monoterpene produced. This shows how simple changes in the production of a single intermediate can result in drastic changes in the metabolic profiles. When compared to heterodimeric GPP synthase, transcripts for farnesyl diphosphate synthase which is responsible for the formation of farnesyl diphosphate precursor for sesquiterpenes, is expressed around 4 times less in PGT. Apart from low MVA pathway, low levels of FPP synthase transcripts might also contribute to the reduced sesquiterpene production in mint PGT.

### Functional characterization of Terpene Synthases (TPS) from spearmint

In plants, specific TPSs are responsible for the synthesis of various terpene molecules from the common precursors. Our transcriptome data provides a rich resource for identifying and functionally characterizing new TPSs from spearmint. From our enriched unigenes, we found 16 that were identified as terpene synthases; all of them were more than 1 kb and 10 of them were encoding full-length open reading frames (ORFs). We found TPSs annotated as limonene synthase, (E)-β-farnesene synthase, bicyclogermacrene synthase and cis muuroladiene synthase being preferentially expressed in PGT. However, the exact functional annotation of a new TPS requires activity characterization of the recombinant protein. In mint species, to our knowledge limonene synthase from spearmint [52], (*E*)-β-farnesene synthase from peppermint [42] and cis-muuroladiene synthase from black peppermint [53] have been previously characterised with respect to their functions. From our RNA seq data we chose to characterize two unannotated full-length TPS. Phylogenetic comparison (Additional file 7) showed that both MsTPS1 and MsTPS2 belonged to the TPS-a subfamily of angiosperm sesquiterpene synthases. The main sesquiterpenes identified by GC-MS analysis in our spearmint variety were (E)-β-farnesene, β-caryophyllene, α-caryophyllene (Humulene), cis murola-3-5 diene, β-copaene, bicyclogermacrene and bicyclosesquiphellandrene.

**Figure 7:**
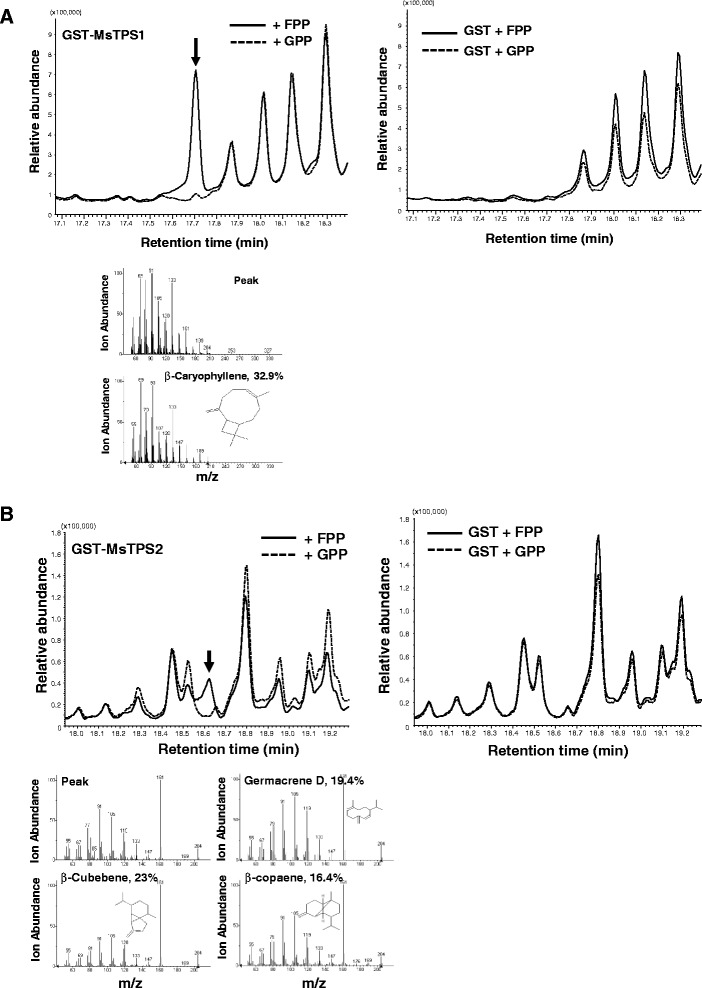
***In vitro*** **enzymatic assays of recombinant MsTPSs.** GST-tagged MsTPS recombinant enzymes were purified by glutathione-based affinity chromatography and used for *in vitro* assays with GPP or FPP as substrate. The final products were analysed by GC-MS. The peaks marked with an arrow in the GC traces were compared with the reference of the mass spectra library. Mass spectra for the peaks formed with FPP are shown at bottom of figure. m/z, mass-to-charge ratio, **(A) **Left panel, β-caryophyllene formation by GST-MsTPS1 with FPP; Right panel, the control GC-MS analyses of GST with GPP or FPP. **(B)** Left panel, β-cubebene/Germacrene D or β-copaene formation by GST-MsTPS2 with FPP; Right panel, the control GC-MS analyses of GST with GPP or FPP.

To determine MsTPS1 and MsTPS2 enzymatic activities, the full-length open reading frame encoding these enzymes were overexpressed in *E.coli*, purified and used for in vitro assays with GPP or FPP as substrate. In the presence of FPP MsTPS1 catalysed the formation of β-caryophyllene in vitro (Figure 7A) whereas MsTPS2 produced a peak from FPP that was identified as one of β-cubebene/Germacrene D/β-copaene by GCMS (Figure 7B). β-copaene was observed in our mint leaf GC-MS data suggesting that MsTPS2 is most likely to be β-copaene synthase. Both the TPSs failed to produce a peak with GPP as substrate (Figure 7A and B). Thus, our in vitro studies identified them to be sesquiterpene synthases. Furthermore, transient *Agrobacterium tumefaciens*-mediated plant expression [54] was used to investigate the terpenes produced by *MsTPS1* and *MsTPS2 in planta*. Both *MsTPS1* and *MSTPS2* under the control of a 35S promoter were transiently expressed in *N. benthamiana* leaves by *Agrobacterium*-mediated infiltration. The compounds were analysed 3 dpi (days post-infiltration) by GC-MS. Both TPSs failed to form any new peak when observed by GC-MS. Studies have shown that overexpressing enzyme 3-Hydroxy-3-Methylglutaryl Coenzyme A Reductase (HMGR), a rate limiting step of the mevalonate pathway increases heterologous plant sesquiterpene production [55]. Accordingly, both the TPSs were coexpressed with HMGR *in planta* to observe the production of sesquiterpenes. MsTPS1 with HMGR produced β-caryophyllene as the major peak and α-caryophyllene and caryophyllene oxide as minor peaks Additional file 8. MsTPS2 even with HMGR failed to produce any new peaks *in planta* suggesting that the compound formed by MsTPS2 might be further metabolised endogenously by the plant (data not shown).

Sesquiterpene synthesis is thought to take place in the cytoplasm while monoterpene synthesis is believed to occur in plastids. Since our biochemical characterization shows that *MsTPS1* and *MsTPS2* are sesquiterpene synthases, we examined subcellular localization of these proteins in transient studies in tobacco leaves. YFP-tagged MsTPSs were transiently expressed in *N. benthamiana* leaf cells by *Agrobacterium*-mediated infiltration and visualized 3 dpi using the YFP channel of a confocal microscope. Both the MsTPSs showed cytoplasmic localization (Additional file 9) and their sequence analysis also showed lack of plastid targeting sequences which further affirms that these TPSs are sesquiterpene synthases. Therefore, either by sequence similarity or by functional characterization we were able to identify all the major terpene synthases that are responsible for the formation of major spearmint essential oil components.

### Spearmint PGTs as plants chemical defense organs

Many of the secondary metabolites produced by glandular trichomes play a role in plant defence. Majority of them fall into the category of terpenes, phenylpropenes, flavonoids, methyl ketones, acyl sugars and defensive proteins. Apart from having a rich terpene pathway, spearmint PGT also shows presence of transcripts that are involved in the production of different secondary metabolites that may have a role in plant defense. Transcripts encoding enzymes for phenylalanine ammonia lyase, cinnamate 4-hydroxylase, and 4-coumarate CoA-ligase were seen expressed in PGT. These enzymes are involved in phenylpropanoid production. Presence of a variety of small molecular weight phenylpropanoids like caffeic, rosmarinic and ferulic acids has been detected in leaves of different mint germplasm [56]. Transcripts similar to Caffeate O-methyltransferase, an enzyme required for the conversion of caffeic acid into ferulic acid was preferentially expressed in PGT. Chalcone flavanone isomerase an enzyme in the flavonoid pathway in plants was also preferentially expressed in PGT. Unigenes encoding transcripts similar to plant invertase/pectin methylesterase inhibitors were highly expressed in PGT which are important to defend against plant pathogens [57,58]. All the above transcripts were verified by qRT-PCR (Additional file 4).

Oxylipins are large family of biologically active oxidized fatty acid-derivatives that are important for plant defense responses. They are produced mainly by the combined action of lipases, lipoxygenases (LOXs) and members of cytochrome P450 (CYP74) family specialized in metabolizing fatty acids hydroperoxides (HPs). HPs form the initial substrates for different branches of the oxylipin pathway [59-62]. We found enzymes involved in oxidation of fatty acids expressed more in PGTs. Transcripts similar to phospholipase A, lipoxygenase and allene oxide synthase and allene oxide cyclase were enriched in PGT. Allene oxide synthase and allene oxide cyclase are members of the cytochrome P450 (CYP74) family and are involved in the LOX pathways [63,64]. All the above mentioned transcripts showed a minimum of 8 times enrichment in PGT and were confirmed by qRT-PCR. However to understand the role of these enzymes in plant defence and exact nature of oxylipins formed by them, further characterization is essential, especially in terms of their positional specificity and substrate specificity. Plants possess both basal and inducible mechanisms to defend themselves against pathogens. The transcriptome data reflects the basal or constitutive state of defense mechanism existing in spearmint PGT. The changes in transcript abundance in all the above mentioned genes on stress induction will provide a better understanding of how PGT act as chemical defence organs.

## Conclusions

Availability of extensive genome resources in non-model plant *Mentha* is scarce. This is the first attempt at the de novo sequencing and assembly of transcriptomes derived from various tissues of *Mentha spicata* using NGS. Comparison of PGT and leaf-PGT led to the identification of 3,919 differentially expressed unigenes. Analysis of these unigenes provides an insight into the gene expression pattern and biological processes active in spearmint PGT. Further identification of various unknown PGT specific unigenes should facilitate new gene discovery. Our transcriptome data provides essential information for future genetic studies in spearmint. It will also help in developing strategies to engineer the terpene metabolic process that can be further extrapolated to other commercially important plants similar to mint where no genomic resources are available.

## Methods

### Plant material, PGT and RNA isolation

Commercial spearmint variety was grown in green house under natural light conditions. 1–2 cm leaves were collected in ice cold imbibition buffer similar to described in [9] in a 50 ml falcon tube. After 1 hour of soaking, the leaves (approximately 3 g) were transferred to a fresh falcon tube containing the extraction buffer as described in [9]. Trichomes were isolated by glass bead abrasion method. Glass beads (Sigma 425–600 μm) were added to the falcon tube and vortexed for 30 sec twice with a 1 min resting period on ice. Then the mixture was carefully passed through cell strainer (100 μM) to remove the cell wall debris and hairy trichomes and the flow through collected. This was again sieved using 40 μM cell strainer to allow passage of capitate glandular trichome and collection of PGT above. The accumulated PGT on top of 40 μM strainer was washed in isolation buffer few times and finally collected using RNAase free water into a 1.5 ml centrifuge tube. The collected PGT was then spun and excess water removed and immediately frozen in liquid nitrogen for further use. We randomly counted around 500 isolated PGT to get an estimate on sample purity and found 26 hair-like trichomes, mostly broken, and 4 CPT indicating a good purity level of isolated PGT. Same stage leaves were brushed in imbibition buffer to remove trichomes and checked under dissection microscope. Total RNA was extracted from PGT, leaf and leaf-PGT using the Spectrum™ Plant total RNA kit from Sigma. The quality of RNA was checked by measuring the ratio of OD_260_ to OD_280_ and the integrity was assessed by measuring the RNA Integrity Number (RIN) number using Agilent 2100 bioanalyser.

### Sequencing and assembly

The RNA libraries were prepared using the TruSeq RNA Sample Preparation Kits v2, set A (RS-122-2001, Illumina Inc.) according to manufacturer’s instructions. The quality and size of cDNA libraries for sequencing were checked using the Agilent 2200 TapeStation system (Agilent Inc.). The libraries were run on single lanes for 100 cycles (paired-end) on Hiseq^™^ 2000 (illumine Inc.), individually. Raw reads were analysed by FastQC [65] for their quality and found to high quality reads with Q > 20. The Trinity method [20] was used for de novo assembly of the raw reads to generate unigenes. Functions of the unigenes were annotated based on sequence similarities to sequences in the public nr database (National Centre for Biotechnology Information) and also the protein sequence databases from *Arabidopsis thaliana*, *Vitis vinifera* and *Oryza sativa*. The GO terms were retrieved by Trinote from the Gene Ontology database [20].

### Preparation of recombinant proteins and *in vitro* enzyme assay

The full-length cDNAs of *MsTPS1* and *MsTPS2* were amplified with the following primer sets from PGT derived cDNA. *MsTPS1*: 5′-CACCATGGAAATTCCTGCACCGGTTTCGGCTTA-3′ and 5′-AACTGTTAGGGGATCAACGAGTATGGATTTGATC-3′; and for *MsTPS2*: and 5′- 5′CACCATGGCTGAAATCTGTGCGTCGGCTGCT-3′ and 5′ GTGCAGGGGATCTACGAGCACGGATTGAAT-3′. To construct the vectors for the production of recombinant GST-tagged proteins, the PCR-amplified *MsTPS1* and *MsTPS2* cDNAs were inserted into pGEX-4 T-1 (GE Healthcare Life Sciences) to generate GST-MsTPS1 and GST-MsTPS2, respectively. Both these constructs were transformed into *E.coli* BL21-CodonPlus (DE3)-RIPL (Stratagene), and treated with 0.2 mM isopropyl 1-thio-β-D-galactopyranoside (IPTG) at 20°C for overnight to induce GST-tagged protein expression. The harvested cell pellets were resuspended in lysis buffer (20 mM Tris, pH 7.4, 150 mM NaCl, 10 mM β-mercaptoethanol, 1 mM phenylmethylsulfonyl fluoride, and protease inhibitors cocktail) and broken by sonication. The clarified lysate was collected by centrifugation and incubated with glutathione Sepharose 4B resin (GE Healthcare Life Sciences) at 4°C overnight. Proteins bound to glutathione Sepharose 4B resin were washed with the purification buffer, eluted from the column with 10 mM glutathione and dialyzed against a buffer containing 25 mM HEPES, pH 7.5, 100 mM KCl, 1 mM DTT, and 5% glycerol.

For *in vitro* enzyme assay for terpene synthase activity, 10 μg of recombinant protein was used in final volume of 500 μl of reaction buffer (25 mM HEPES, pH 7.5, 100 mM KCl, 7.5 mM MgCl_2_, 5 mM DTT, and 5% glycerol) with 10 μg of substrate (farnesyl diphosphate, FPP or geranyl diphosphate, GPP; Sigma). Overlaid 250 μl of hexane to trap volatile products and incubated at 30°C for 2 h. Extracts were analysed by GC-MS (Agilent).

### *In vivo* characterization of *MsTPS1*

The full length cDNA encoding Arabidopsis 3-hydroxy-3-methylglutaryl coenzyme A reductase (*AtHMGR1*, At1g76490) was amplified using two specific primers, AtHMGR-F-XbaI (5′-AACTCTAGAATGAAGAAAAAGCAAGCTGGTCCCCAACAGA-3′) and AtHMGR-R-AscI primers (5′-AAAGGCGCGCCTGTTGTTGTTGTTGTCGTTGTCGTT-3′). The PCR-amplified product was digested by *Xba*I and *Asc*I, and cloned into pCAMBIA1300-3HA. *In vivo* characterization was done in *Nicotianana benthamiana* leaves by *Agrobacterium*-mediated infiltration. Initially *Agrobacterium* strain harbouring *MsTPS1* was used alone and then strain carrying AtHMGR1 was mixed with MSTPS1 strain and coinfiltrated. Overnight cultures of *Agrobacterium* grown at 28°C were harvested. It was resuspended to a final concentration to an absorbance of 1.0 at 600 nm in a solution containing 10 mM MgCl_2_, 10 mM MES pH 5.6 and 100 μM acetosyringone. After 2 hour incubation at room temperature, the *Agrobacterium* mixture was injected into *N. benthamiana* leaves using a needleless syringe. Infiltrated plants were incubated in the growth chamber at 24°C for 3 days or 6 days. Three to four infiltrated leaves were taken for GC-MS analysis.

### GC-MS analysis method

For extraction about 4–6 leaves were ground to a fine powder using liquid nitrogen and the powder homogenised in 500 μl ethyl acetate including 1 μl (10 mg/ml) of camphor as internal standard and incubated at least 2 h at room temperature with shaking. This mixture was centrifuged for 5 min at 12,000 rpm. The top organic layer was transferred to a new tube and dehydrated using anhydrous Na_2_SO_4._ The samples were analysed using GCMS (Agilent Technologies 7890A with 5975C inert Mass-spe-ctro Detector with tripe axis detector). 2 μl of samples were injected and separation was achieved with a tem-perature program of 50°C for 1 min and increased at a rate of 8°C/min to 300°C and held for 5 min, on a 30 m HP-5 MS column (Agilent Technologies).

### Subcellular localization of TPSs-

The full-length cDNAs of *MsTPS1* and *MsTPS2* were amplified with the following primer sets from PGT derived cDNA. *MsTPS1*: 5′-CACCATGGAAATTCCTGCACCGGTTTCGGCTTA-3′ and 5′-AACTGTTAGGGGATCAACGAGTATGGATTTGATC-3′; and for *MsTPS2*: and 5′- 5′CACCATGGCTGAAATCTGTGCGTCGGCTGCT-3′ and 5′ GTGCAGGGGATCTACGAGCACGGATTGAAT-3′. Both the cDNAs were cloned into pENTR/D-TOPO vector (Invitrogen), and then transferred into pBA-DC-YFP [66] which contains the CaMV 35S promoter and C-terminal in frame YFP, to generate MsTPS1-YFP and MsTPS2-YFP, respectively. The MsTPS1-YFP or Ms-TPS2-YFP constructs were introduced into *A. tumefaciens* strain GV3101 by electroporation. YFP-tagged MsTPSs were co-expressed with CFP or alone to confirm the cellular localization of MSTPS-YFP. CFP expression was used as a cytoplasmic maker protein. Overnight cultures of *Agrobacterium* grown at 28°C were harvested. It was resuspended to a final concentration to an absorbance of 1.0 at 600 nm in a solution containing 10 mM MgCl_2_, 10 mM MES pH 5.6 and 100 μM acetosyringone. After 2 hour incubation at room temperature, the *Agrobacterium* mixture was injected into *N. benthamiana* leaves using a needleless syringe. Infiltrated tobacco plants were placed in the growth chamber at 24°C for 3 days. Fluorescence signals were detected by a confocal scanning laser microscopy (Carl Zeiss LSM 5 Exciter) with a standard filter set.

### Quantitative real time PCR (qRT-PCR)

The qRT-PCR was employed to validate gene expression pattern of transcriptome analysis. Approximately 1 μg RNA was used for cDNA synthesis with iScript supermix (Bio-rad). The reaction was carried out by incubation for priming at 25°C for 5 min, followed by reverse transcription at 42°C for 30 min and inactivation at 85°C for 5 min. cDNA was stored at −20°C till further used. The qRT-PCR reactions were performed in 384-well PCR plate using ABI PRISM 900HT real-time PCR system and KAPA SYBR fast master mix (KAPA Biosystems).PCR reactions were performed using 0.3 μl of the cDNA in a total of 5 μl reaction volume and cycling profile was 95°C for 10 min, 40 cycles of 95°C for 15 s and 60°C for 60 s. After thermal cycles, the dissociation analysis (melting curve) was carried out to confirm specific amplification of PCR reaction. All reactions were performed in triplicate with three biological replicates, including non-template control. The threshold cycle (C_T_) value of gene is the cycle number required for SYBR Green fluorescence signal to reach the threshold level during the exponential phase for detecting the amount of accumulated nucleic acid [67]. In current study, elongation factor 1 (*ef1*) was used as internal control, due to its stable expression in plant [68] and it also showed similar expression levels in all the tissues in our RNA seq data. Comparative delta C_T_ values of target genes to *ef1* were taken as relative expression among different tissues. The amount of target gene, normalized to *ef1* gene, was calculated by 2^-(C^_T_^target gene-C^_T_^*ef1*)^. Results were represented as mean ± SD. The unigene names and sequence of primers used are listed in Additional file 10.

### Availability of supporting data

The raw RNA seq data supporting the result of this article is available in the DDBJ: DNA Data Bank of Japan, with accession number is: DRA001856 (http://trace.ddbj.nig.ac.jp/DRASearch/submission?acc=DRA001856).

## Additional files

Additional file 1:
**Tissues from which RNA were isolated.** a, isolated PGT; b, leaf-PGT; c, leaf. Additional file 2:
**Quality of reads and statistics of sequencing.**
Additional file 3:
**Differentially expressed genes in PGT.**
Additional file 4:
**qRT-PCR analyses of described unigenes.** DXS: 1-deoxy-D-xylulose-5-phosphate (DXP) synthase; DXR: DXP reductoisomerase, MCT:MEP cytidyltransferase, CMK:4-(cytidine 5- diphospho)-2-*C*-methyl-D-erythritol kinase MCS: 2-C-methyl-D-erythritol 2,4-cyclodiphosphate (ME-2,4cPP) synthase, HDS: 1-hydroxy-2-methyl-2-butenyl 4-diphosphate (HMBPP) synthase, HDR: HMBPP reductase, IPPI : Isopentenyl diphosphate (IPP,C5) Delta-isomerase, GPPS: geranyl diphosphate synthase, LS: limonene synthase, L6OH: Limonene-6-hydroxylase, CD: carveol dehydrogenase, LTP: Lipid transfer protein. Additional file 5:
**Subset of cell wall related unigenes more abundant in PGT.**
Additional file 6:
**Expression levels of transcripts involved in MVA pathway.** The number in green represents the expression level of a particular unigene in PGT (log2 of estimate abundance of transcripts by RSEM value). The number in red represents the fold change in expression level when compared to leaf-PGT (log2 fold change between PGT and leaf-PGT). In cases of enzymes with more than one unigene, the unigene with the highest abundance was taken into consideration. The number in brackets represents the number of unigenes identified for each enzyme in the pathway. Additional file 7:
**Phylogenetic analysis of full-length MsTPS1 and MsTPS2 to other plant terpene synthases.** The neighbour-joining tree was created using MEGA5.2 program from an alignment of *Mentha piperita* (*E*)-β-farnesene synthase (AF024615), *Lycopersicon esculentum* germacrene C synthase (AF035630), *Salvia officinalis* 1,8-cineole synthase (AF051899), *Mentha spicata* 4S-limonene synthase (L13459), *Perilla citriodora* limonene synthase (AF241790), *Salvia officinalis* (+)-bornyl diphosphate synthase (AF051900), *Salvia officinalis* (+)-sabinene synthase (AF051901), *Perilla frutescens* linalool synthase (AF444798), *Citrus limon* (+)-limonene synthase 1 (AF514287), *Arabidopsis thaliana* myrcene/ocimene synthase (AF178535), *Artemisia annua* (3R)-linalool synthase (AF154124), *Antirrhinum majus* nerolidol/linalool synthase 1 (EF433761), *Antirrhinum majus* (*E*)- β -ocimene synthase (AY195607), *Solanum lycopersicum* copalyl diphosphate synthase (AB015675), *Cucurbita maxima* copalyl diphosphate synthase (AF049905), *Zea mays* terpene synthase 1 (AF529266), *Cucurbita maxima* copalyl diphosphate synthase 1 (AF049905), *Abies grandis* δ-selinene synthase (U92266), *Abies grandis* (−)-4S-limonene synthase (AF006193), *Abies grandis* pinene synthase (U87909), *Abies grandis* terpinolene synthase (AF139206), *Abies grandis* myrcene synthase (U87908), *Picea abies E*-α-bisabolene synthase (AY473619), *Cichorium intybus* germacrene A synthase short form (AF498000), *Lactuca sativa* germacrene A synthase LTC1 (AF489964), *Solidago canadensis* germacrene D synthase (AJ583447), *Solidago canadensis* germacrene A synthase (AJ304452), *Artemisia annua* β-caryophyllene synthase QHS1 (AF472361), *Artemisia annua* amorpha-4,11-diene synthase (JQ319661), *Artemisia annua* (*E*)-β-farnesene synthase (AY835398), *Arabidopsis thaliana* copalyl diphosphate synthase (NM_116512), *Arabidopsis thaliana* ent-kaurene synthase GA2 (AF034774), *Stevia rebaudiana* copalyl pyrophosphate synthase (AF034545), *Stevia rebaudiana* kaurene synthase (AF097310). The scale bar indicates the number of amino acid substitutions per site. Five TPS subfamilies, a to e are based on the taxonomic distribution of the TPS families [69]. Additional file 8:
**GC-MS data generated from** ***in vivo*** **characterization of MsTPS1.** MsTPS1 with or without HMGR was transiently expressed in *N. benthamiana* leaves by *Agrobacterium*-mediated infiltration. The compounds were analysed 3 dpi (days post-infiltration) by GC-MS. Numbered peaks were identified by the reference of the mass spectra library and the mass spectra of compounds are shown at right side. Additional file 9:
**Subcellular localization of** ***MsTPS1*** **and** ***MsTPS2*** **in** ***N. benthamiana*** **leaf.** **(A)** YFP-tagged MsTPSs were transiently expressed in *N. benthamiana* leaf cells by *Agrobacterium*-mediated infiltration and visualized 3 dpi using YFP channel of a confocal microscope. **(B)** YFP-tagged MsTPSs were co-expressed with CFP to confirm the cytoplasmic localization of MSTPS-YFP. CFP expression is used as a cytoplasmic maker protein. CFP: CFP channel image, YFP: YFP channel image, Auto: chlorophyll auto fluorescence, Light: light microscope image, Merged: merged image between Light and YFP. Additional file 10:
**Primer sequences used for qRT-PCR analysis.**

## Abbreviations

PGT: Peltate glandular trichomes
TPS: Terpene synthases
EST: Expressed sequence tags
GC-MS: Gas chromatography–mass spectrometry
RSEM: RNA-seq by Expectation-Maximization
LTP: Lipid transfer proteins
GPT: Glc6P–phosphate translocator
NTT: Nucleotide transporter
MVA: Mevalonate pathway
MEP: 2-Cmethyl-D-erythritol-4-phosphate
IPP: Isopentenyl diphosphate
DMAPP: Dimethylallyl diphosphate
GPP: Geranyl diphosphate
FPP: Farnesyl diphosphate
GGPP: Geranylgeranyl diphosphate
DXS: 1-deoxy-D-xylulose-5-phosphate synthase
DXR: 1-deoxy-D-xylulose 5-phosphate reductoisomerase
MCT: 2-*C*-methyl-D-erythritol 4-phosphate cytidylyltransferase
CMK: 4-(cytidine 5-diphospho)-2-*C*-methyl-D-erythritol kinase
MCS: 2-C-methyl-D-erythritol 2,4-cyclodiphosphate synthase
HDS: 1-hydroxy-2-methyl-2-butenyl 4-diphosphate synthase
HDR: 1-hydroxy-2-methyl-2-butenyl 4-diphosphate reductase
IPPI: Isopentenyl diphosphate isomerase
GPPS: Geranyl diphosphate synthase
LS: Limonene synthase
L6OH: Limonene-6-hydroxylase
CD: Carveol dehydrogenase
ORF: Open reading frame
dpi: Days post-infiltration
HMGR: Hydroxy-3-Methylglutaryl Coenzyme A Reductase
LOX: Lipoxygenases
