# Supplementary figures and images for: Next generation sequencing unravels the biosynthetic ability of Spearmint (Mentha spicata) peltate glandular trichomes through comparative transcriptomics
Source: BMC Plant Biol. 2014 Nov 1;14:292. doi: 10.1186/s12870-014-0292-5 (PMC4232691; doi:10.1186/s12870-014-0292-5)

## Slide 1
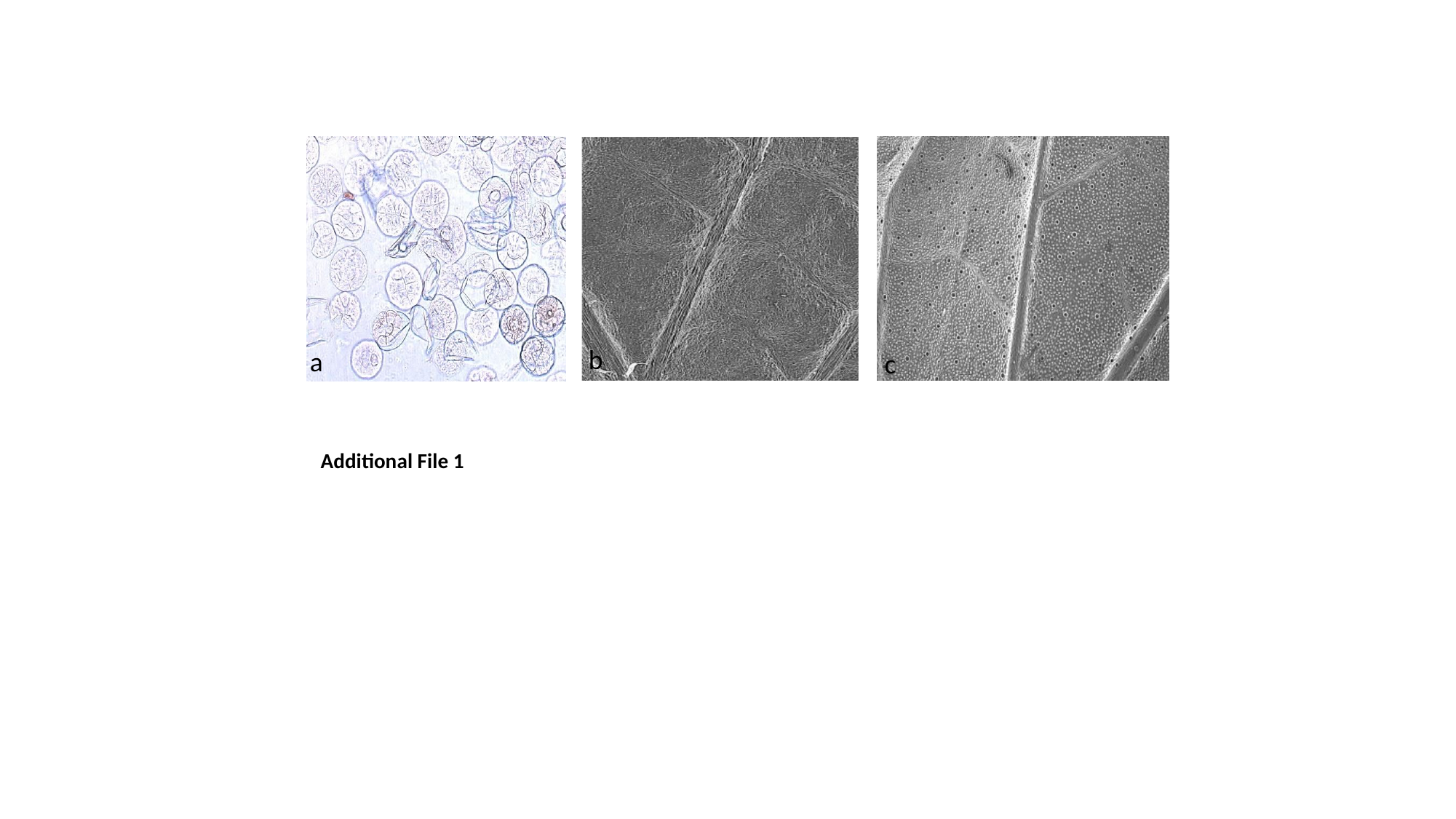

b
Roots
c
a
Additional File 1

Supplement: Additional file 1: — Tissues from which RNA were isolated. a, isolated PGT; b, leaf-PGT; c, leaf. [file 12870_2014_292_MOESM1_ESM.pptx]

## Slide 1
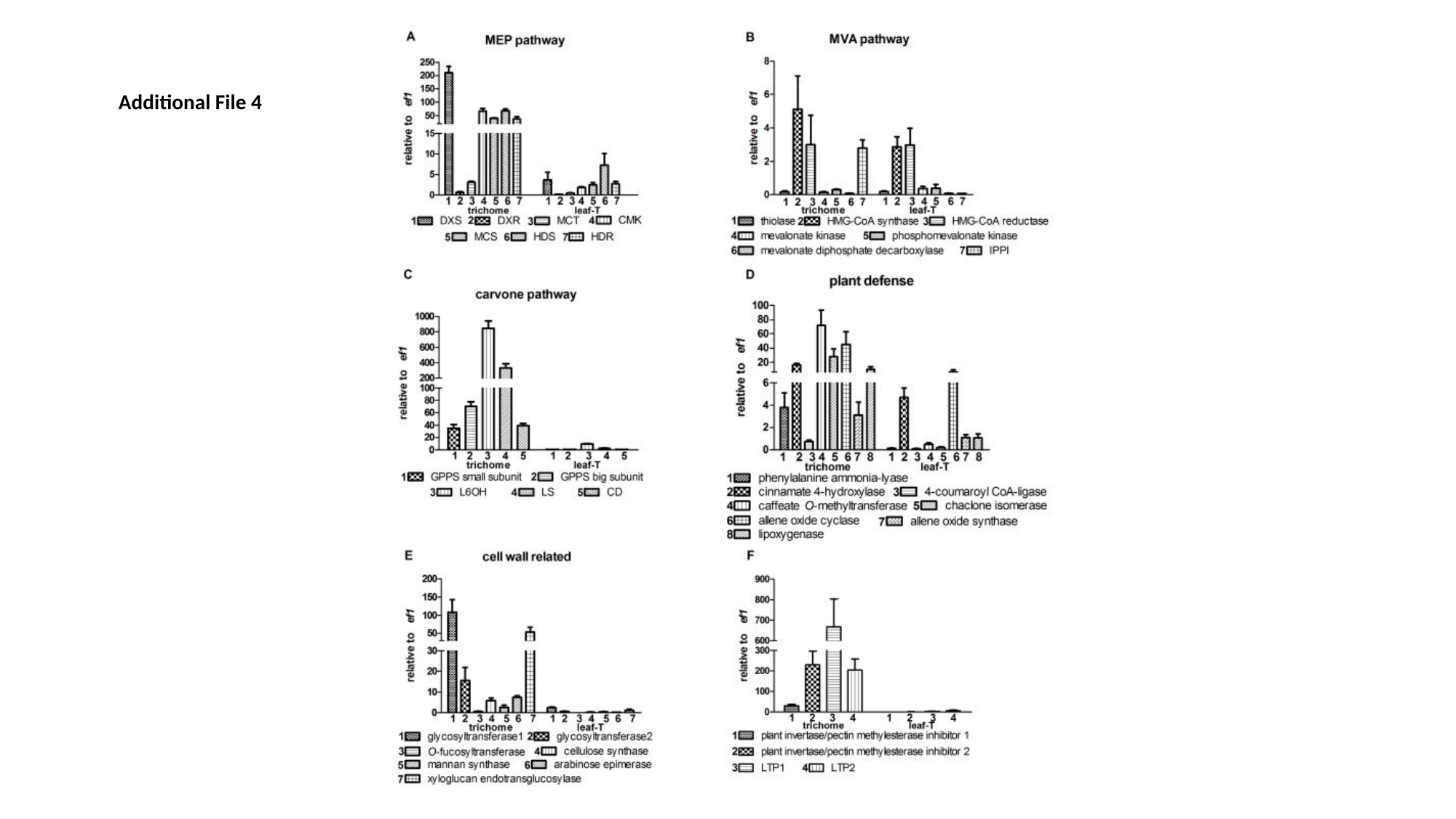

Additional File 4

Supplement: Additional file 4: — qRT-PCR analyses of described unigenes. DXS: 1-deoxy-D-xylulose-5-phosphate (DXP) synthase; DXR: DXP reductoisomerase, MCT:MEP cytidyltransferase, CMK:4-(cytidine 5- diphospho)-2-C-methyl-D-erythritol kinase MCS: 2-C-methyl-D-erythritol 2,4-cyclodiphosphate (ME-2,4cPP) synthase, HDS: 1-hydroxy-2-methyl-2-butenyl 4-diphosphate (HMBPP) synthase, HDR: HMBPP reductase, IPPI : Isopentenyl diphosphate (IPP,C5) Delta-isomerase, GPPS: geranyl diphosphate synthase, LS: limonene synthase, L6OH: Limonene-6-hydroxylase, CD: carveol dehydrogenase, LTP: Lipid transfer protein. [file 12870_2014_292_MOESM4_ESM.pptx]
