# Supplementary material for: Next generation sequencing unravels the biosynthetic ability of Spearmint (Mentha spicata) peltate glandular trichomes through comparative transcriptomics
Source: BMC Plant Biol. 2014 Nov 1;14:292. doi: 10.1186/s12870-014-0292-5 (PMC4232691; doi:10.1186/s12870-014-0292-5)
Supplement: Additional file 2: — Quality of reads and statistics of sequencing. [file 12870_2014_292_MOESM2_ESM.pptx]

## Slide 1
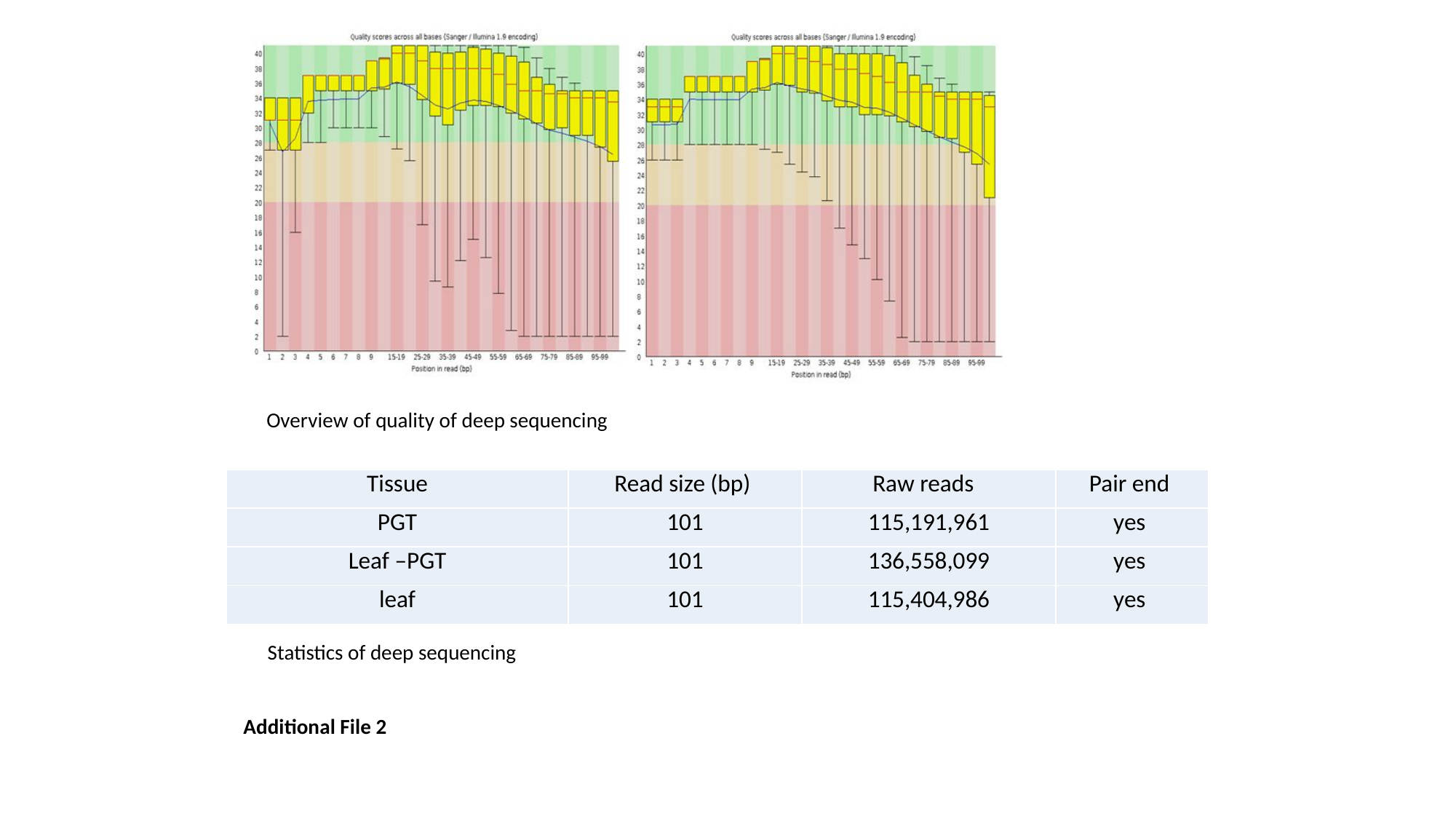

Overview of quality of deep sequencing
| Tissue | Read size (bp) | Raw reads | Pair end |
| --- | --- | --- | --- |
| PGT | 101 | 115,191,961 | yes |
| Leaf –PGT | 101 | 136,558,099 | yes |
| leaf | 101 | 115,404,986 | yes |
Statistics of deep sequencing
Additional File 2
