# Supplementary material for: Next generation sequencing unravels the biosynthetic ability of Spearmint (Mentha spicata) peltate glandular trichomes through comparative transcriptomics
Source: BMC Plant Biol. 2014 Nov 1;14:292. doi: 10.1186/s12870-014-0292-5 (PMC4232691; doi:10.1186/s12870-014-0292-5)
Supplement: Additional file 6: — Expression levels of transcripts involved in MVA pathway. The number in green represents the expression level of a particular unigene in PGT (log2 of estimate abundance of transcripts by RSEM value). The number in red represents the fold change in expression level when compared to leaf-PGT (log2 fold change between PGT and leaf-PGT). In cases of enzymes with more than one unigene, the unigene with the highest abundance was taken into consideration. The number in brackets represents the number of unigenes identified for each enzyme in the pathway. [file 12870_2014_292_MOESM6_ESM.pptx]

## Slide 1
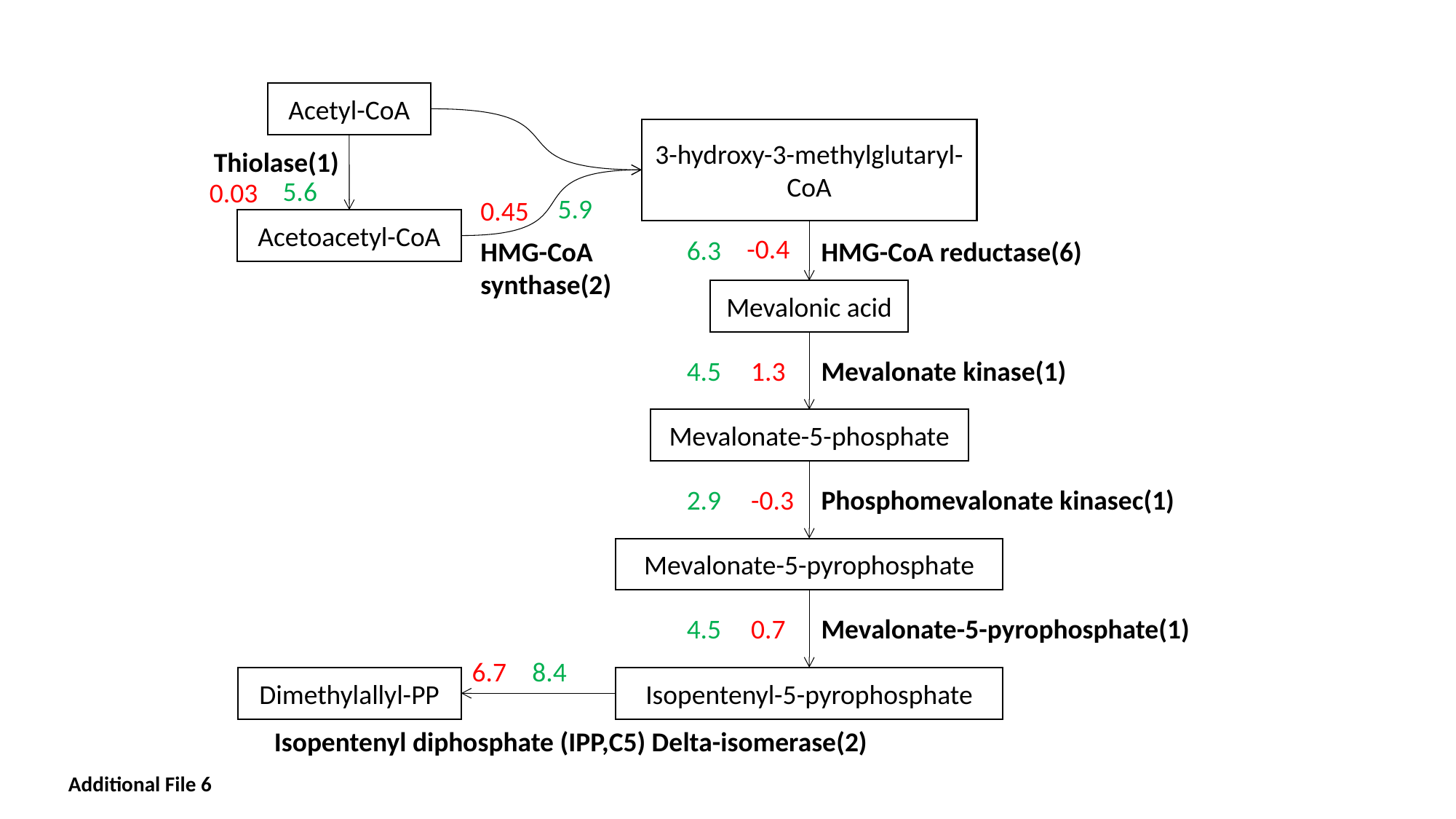

Acetyl-CoA
3-hydroxy-3-methylglutaryl-CoA
Thiolase(1)
5.6
0.03
5.9
0.45
Acetoacetyl-CoA
-0.4
6.3
HMG-CoA reductase(6)
HMG-CoA synthase(2)
Mevalonic acid
4.5
1.3
Mevalonate kinase(1)
Mevalonate-5-phosphate
2.9
-0.3
Phosphomevalonate kinasec(1)
Mevalonate-5-pyrophosphate
4.5
0.7
Mevalonate-5-pyrophosphate(1)
6.7
8.4
Dimethylallyl-PP
Isopentenyl-5-pyrophosphate
Isopentenyl diphosphate (IPP,C5) Delta-isomerase(2)
Additional File 6
