# Supplementary material for: Next generation sequencing unravels the biosynthetic ability of Spearmint (Mentha spicata) peltate glandular trichomes through comparative transcriptomics
Source: BMC Plant Biol. 2014 Nov 1;14:292. doi: 10.1186/s12870-014-0292-5 (PMC4232691; doi:10.1186/s12870-014-0292-5)
Supplement: Additional file 7: — Phylogenetic analysis of full-length MsTPS1 and MsTPS2 to other plant terpene synthases. The neighbour-joining tree was created using MEGA5.2 program from an alignment of Mentha piperita (E)-β-farnesene synthase (AF024615), Lycopersicon esculentum germacrene C synthase (AF035630), Salvia officinalis 1,8-cineole synthase (AF051899), Mentha spicata 4S-limonene synthase (L13459), Perilla citriodora limonene synthase (AF241790), Salvia officinalis (+)-bornyl diphosphate synthase (AF051900), Salvia officinalis (+)-sabinene synthase (AF051901), Perilla frutescens linalool synthase (AF444798), Citrus limon (+)-limonene synthase 1 (AF514287), Arabidopsis thaliana myrcene/ocimene synthase (AF178535), Artemisia annua (3R)-linalool synthase (AF154124), Antirrhinum majus nerolidol/linalool synthase 1 (EF433761), Antirrhinum majus (E)- β -ocimene synthase (AY195607), Solanum lycopersicum copalyl diphosphate synthase (AB015675), Cucurbita maxima copalyl diphosphate synthase (AF049905), Zea mays terpene synthase 1 (AF529266), Cucurbita maxima copalyl diphosphate synthase 1 (AF049905), Abies grandis δ-selinene synthase (U92266), Abies grandis (−)-4S-limonene synthase (AF006193), Abies grandis pinene synthase (U87909), Abies grandis terpinolene synthase (AF139206), Abies grandis myrcene synthase (U87908), Picea abies E-α-bisabolene synthase (AY473619), Cichorium intybus germacrene A synthase short form (AF498000), Lactuca sativa germacrene A synthase LTC1 (AF489964), Solidago canadensis germacrene D synthase (AJ583447), Solidago canadensis germacrene A synthase (AJ304452), Artemisia annua β-caryophyllene synthase QHS1 (AF472361), Artemisia annua amorpha-4,11-diene synthase (JQ319661), Artemisia annua (E)-β-farnesene synthase (AY835398), Arabidopsis thaliana copalyl diphosphate synthase (NM_116512), Arabidopsis thaliana ent-kaurene synthase GA2 (AF034774), Stevia rebaudiana copalyl pyrophosphate synthase (AF034545), Stevia rebaudiana kaurene synthase (AF097310). The scale bar indicat [file 12870_2014_292_MOESM7_ESM.pptx]

## Slide 1
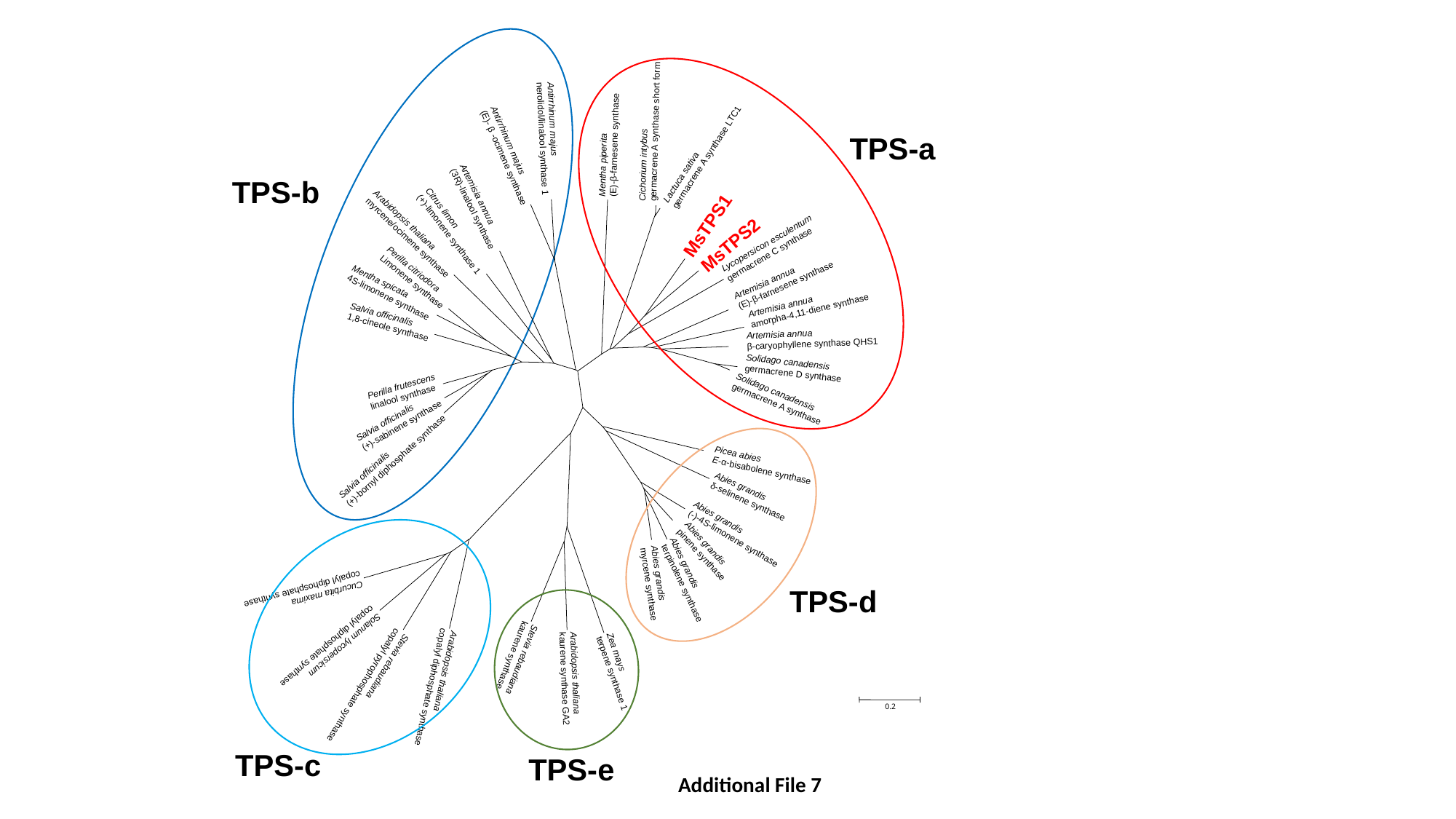

Cichorium intybus
germacrene A synthase short form
TPS-a
Antirrhinum majus
nerolidol/linalool synthase 1
Mentha piperita
(E)-β-farnesene synthase
Lactuca sativa
germacrene A synthase LTC1
Antirrhinum majus
(E)- β -ocimene synthase
TPS-b
Artemisia annua
(3R)-linalool synthase
MsTPS1
Citrus limon
(+)-limonene synthase 1
Arabidopsis thaliana
myrcene/ocimene synthase
MsTPS2
Lycopersicon esculentum
germacrene C synthase
Artemisia annua
(E)-β-farnesene synthase
Perilla citriodora
Limonene synthase
Mentha spicata
4S-limonene synthase
Artemisia annua
amorpha-4,11-diene synthase
Salvia officinalis
1,8-cineole synthase
Artemisia annua
β-caryophyllene synthase QHS1
Solidago canadensis
germacrene D synthase
Perilla frutescens
linalool synthase
Solidago canadensis
germacrene A synthase
Salvia officinalis
(+)-sabinene synthase
Salvia officinalis
(+)-bornyl diphosphate synthase
Picea abies
E-α-bisabolene synthase
Abies grandis
δ-selinene synthase
Abies grandis
(-)-4S-limonene synthase
Abies grandis
pinene synthase
Abies grandis
 terpinolene synthase
Abies grandis
myrcene synthase
TPS-d
Cucurbita maxima
copalyl diphosphate synthase
Solanum lycopersicum
copalyl diphosphate synthase
Stevia rebaudiana
kaurene synthase
Zea mays
terpene synthase 1
Arabidopsis thaliana
kaurene synthase GA2
Stevia rebaudiana
copalyl pyrophosphate synthase
Arabidopsis thaliana
copalyl diphosphate synthase
0.2
TPS-c
TPS-e
Additional File 7
