# Supplementary material for: Next generation sequencing unravels the biosynthetic ability of Spearmint (Mentha spicata) peltate glandular trichomes through comparative transcriptomics
Source: BMC Plant Biol. 2014 Nov 1;14:292. doi: 10.1186/s12870-014-0292-5 (PMC4232691; doi:10.1186/s12870-014-0292-5)
Supplement: Additional file 8: — GC-MS data generated from in vivo characterization of MsTPS1. MsTPS1 with or without HMGR was transiently expressed in N. benthamiana leaves by Agrobacterium-mediated infiltration. The compounds were analysed 3 dpi (days post-infiltration) by GC-MS. Numbered peaks were identified by the reference of the mass spectra library and the mass spectra of compounds are shown at right side. [file 12870_2014_292_MOESM8_ESM.pptx]

## Slide 1
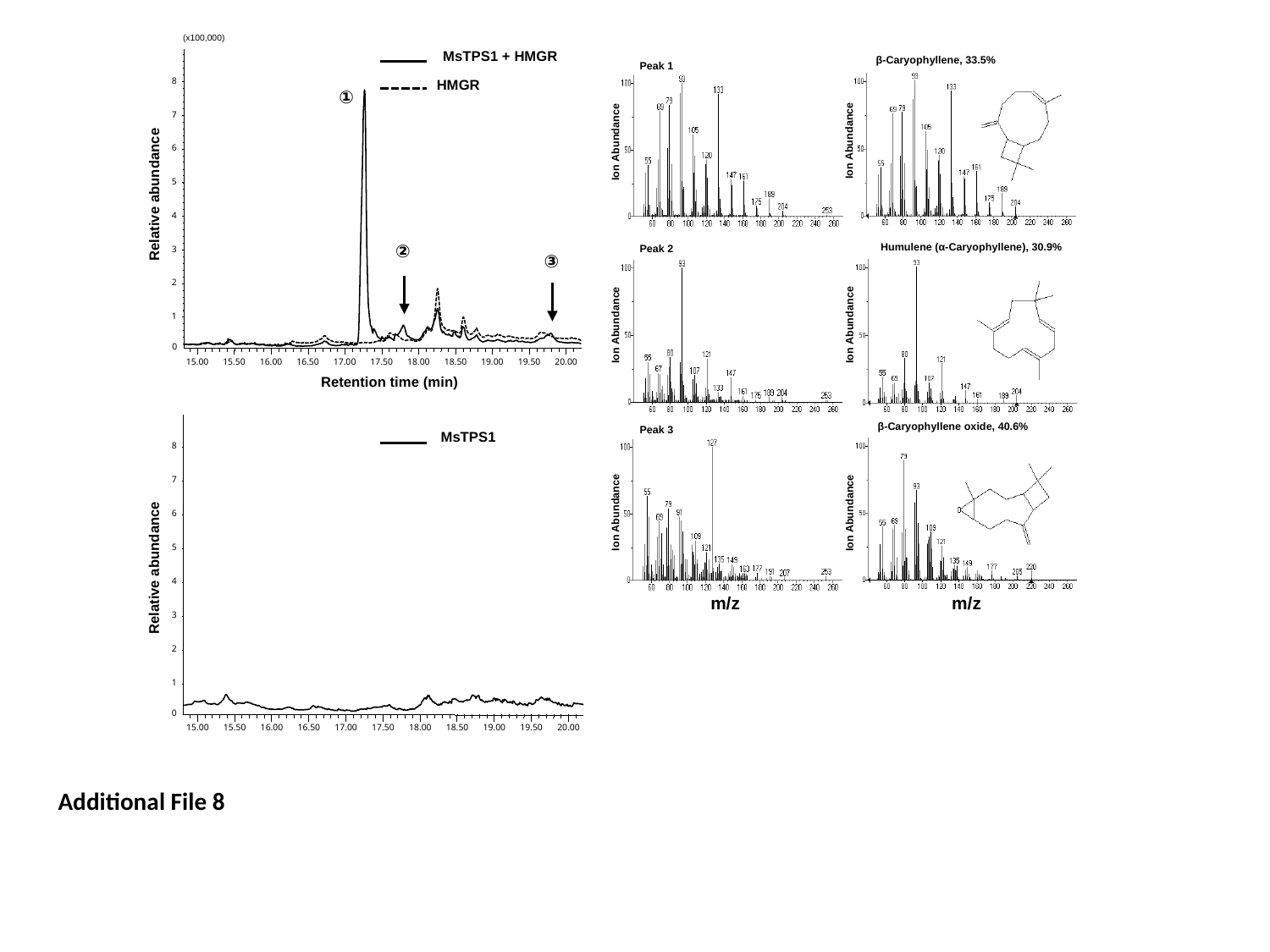

(x100,000)
β-Caryophyllene, 33.5%
MsTPS1 + HMGR
8
7
6
5
4
3
2
1
0
15.00
15.50
16.00
16.50
17.00
17.50
18.00
18.50
19.00
19.50
20.00
Peak 1
HMGR
①
Ion Abundance
Ion Abundance
Relative abundance
②
Humulene (α-Caryophyllene), 30.9%
Peak 2
③
Ion Abundance
Ion Abundance
Retention time (min)
β-Caryophyllene oxide, 40.6%
8
7
6
5
4
3
2
1
0
15.00
15.50
16.00
16.50
17.00
17.50
18.00
18.50
19.00
19.50
20.00
Peak 3
MsTPS1
Ion Abundance
Ion Abundance
Relative abundance
m/z
m/z
Additional File 8
