# Supplementary material for: Next generation sequencing unravels the biosynthetic ability of Spearmint (Mentha spicata) peltate glandular trichomes through comparative transcriptomics
Source: BMC Plant Biol. 2014 Nov 1;14:292. doi: 10.1186/s12870-014-0292-5 (PMC4232691; doi:10.1186/s12870-014-0292-5)
Supplement: Additional file 9: — Subcellular localization of MsTPS1 and MsTPS2 in N. benthamiana leaf. (A) YFP-tagged MsTPSs were transiently expressed in N. benthamiana leaf cells by Agrobacterium-mediated infiltration and visualized 3 dpi using YFP channel of a confocal microscope. (B) YFP-tagged MsTPSs were co-expressed with CFP to confirm the cytoplasmic localization of MSTPS-YFP. CFP expression is used as a cytoplasmic maker protein. CFP: CFP channel image, YFP: YFP channel image, Auto: chlorophyll auto fluorescence, Light: light microscope image, Merged: merged image between Light and YFP. [file 12870_2014_292_MOESM9_ESM.pptx]

## Slide 1
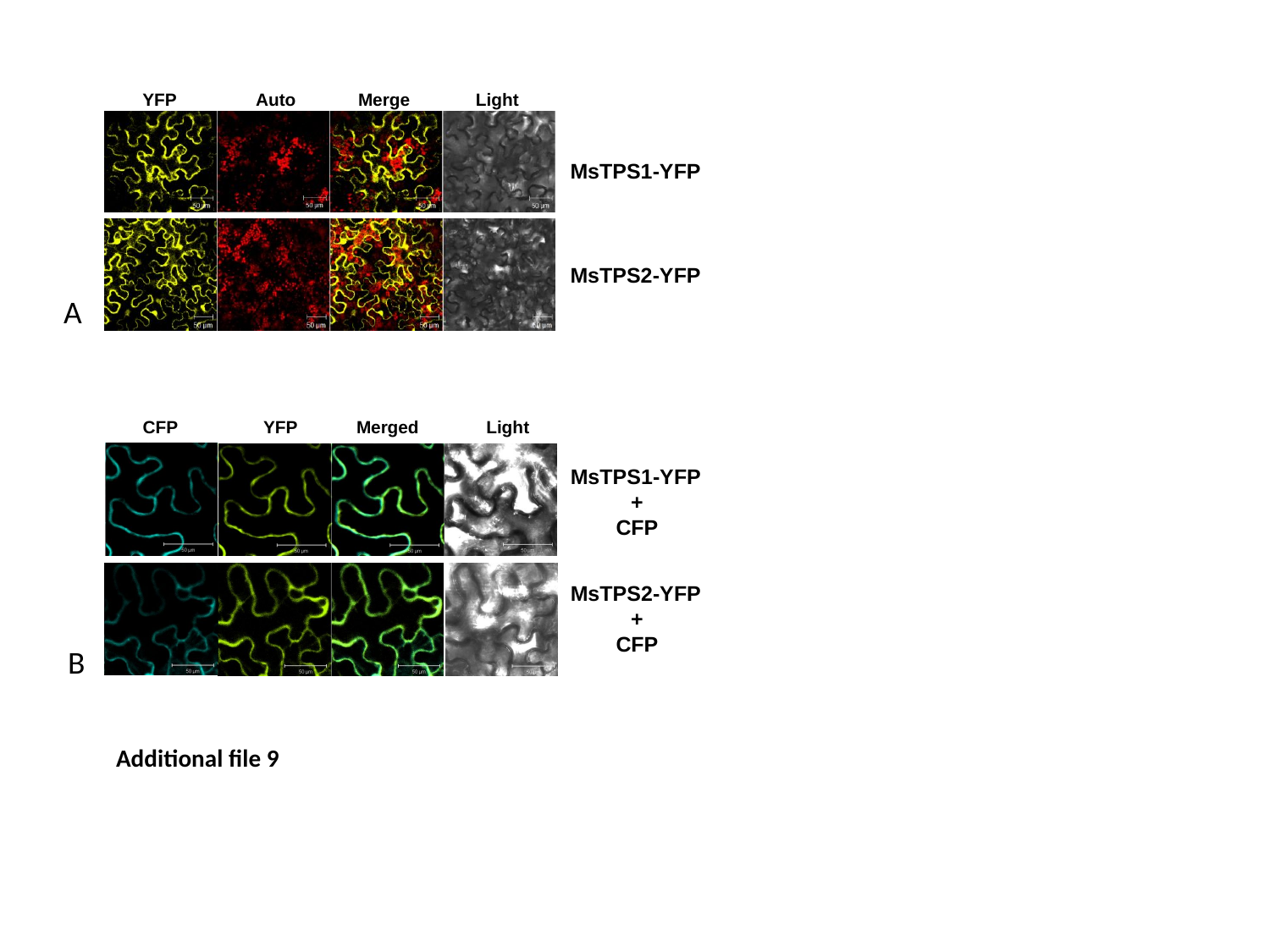

Light
YFP
Auto
Merge
MsTPS1-YFP
MsTPS2-YFP
A
CFP
YFP
Merged
Light
MsTPS1-YFP
+
CFP
MsTPS2-YFP
+
CFP
B
Additional file 9
